# Bichloride of Mercury in Anemia

**Published:** 1889-07

**Authors:** A. M. Cartledge

**Affiliations:** Demonstrator of Anatomy, Kentcky School of Medicine


### BICHLORIDE OF MERCURY IN ANEMIA.

By A. M. CARTLEDGE, M. D.
Demonstrator of Anatomy, Kentcky School of Medicine.

Mercury, when properly given, is without doubt a greater benefit to a larger class of sufferers than any remedy we are acquainted with at the present time. This or that year discovers the enterprising theapeutist in quest of some vegetable substitute for this time-honored agent, but an impartial trial results in a return to the "old love." It may be there is something better in the store-house of nature, but it has not yet been discovered. Because a thing has been with us long we are apt to feel a familiar acquaintance and fail to acquire a thorough knowledge of its properties. It would be well for us to adhere to the maxim to "study that which is good." To Keyes belongs most of the credit of first calling attention to the small doses of mercury in the treatment of syphilis. This was an advance step of great importance, as it raised the profession from the position of doing syphilitic subjects a great injury by treatment to one of imparting the greatest benefits. I have elsewhere, in a paper on mercury in syphilis, spoken at length on this subject. What is desired to especially call attention to in this paper is the wonderful potency of mercury, more particularly the protochloride, as a tonic and curative agent in most forms of anemia. I am aware the explanation of this will appear to most physicians to be the specific taint which underlies so many of these cases. However, I beg to believe that it exercises its influence for good in cases when syphilis by heredity, remote or otherwise, is no factor in the case. Admittedly, the minute pathology of anemia is obscure, many times depending on organic lesions of varied character and situated in different organs. The evidence of anemia, viz., impoverished red blood cells or diminution of red blood cells, does not enlighten us as to the cause of this phenomena.

The shortest as well as the most accurate way of estimating the virtue of mercury in anemia seems to be, first, to fix in our minds what we know the salts of mercury is capable of doing when introduced in the system; second, to study many of the organic lesions, together with their pathological anatomy, which are secondarily, at least, causative elements of anemia; third, to make the application.

Under the first head comes the known power of small quantities of mercury to cause absorption of lymph deposits anywhere in the system, probably by its stimulation of the glandular system, more probably by relieving the gland structures of embarrassment to action, the impediment being micro-organisms which irritate the walls of afferent and efferent ducts, producing engorgements—the mercury acting as a germicide. Its known action outside the body, together with its influence on morbid processes in the body, does more to strengthen the germ theory of disease than any one corroborative fact.

Take these properties alone, viz., its power to cause lymph absorption and its influence in relieving glandular engorgement, whether of the great glands, as the liver, kidneys, etc., or the lymphatic system, and I dare say nearly every pathologist will agree that you have an agent indicated in nine out of every ten cases of anemia. The deduction is, that while iron has its sphere in feeding the impoverished red cell—and we have abundant proof that it does—and while the salts of Peruvian bark and arsenic do meet specific causes of amenia, yet the presence of the deleterious influences soon produce structural changes which are most effectually met by mercury. Of course it maintains pre-eminence as a specific agent in syphilis.

This article would be more incomplete without a word as to the form of administration for the tonic and alterative effect. While I must express a preference for the proto-iodide in the so-called primary and secondary stages of syphilis, I think, where impoverishment of the red corpuscles has taken place, or in anemia without syphilis, the bichloride is the preparation to use. The union with the hydrochloric acid seeming to greatly increase the tonic effect, I often give it in doses as small as the $\frac{1}{64}$ of a grain well diluted on a full stomach. A most excellent combination is that of the protochloride of iron as prepared by Messrs. Renz & Henry, of this city, under the name elixir of three chlorides.

Take the anemia of females, the subject of disease connected with the organs of generation, and I know of no one constitutional remedy the equal of this drug. Nearly all of these cases are the subject of lymph deposits and ovarian congestion, which is best met by an agent which so decidedly facilitates healthy gland action. In the chlorosis which is so often a manifestation of struma the bichloride of mercury with iron will often effect a cure where iron alone fails. We appreciate the great good mercury does, especially as calomel, in relieving acute glandular engorgement. What I think we need most to be impressed with is its great virtue in relieving those often obscure and chronic obstructions to gland action which exert so potent an influence for evil in the economy.—*American Practitioner.*

